# Systemic CD8^+^ T Cell-Mediated Tumoricidal Effects by Intratumoral Treatment of Oncolytic Herpes Simplex Virus with the Agonistic Monoclonal Antibody for Murine Glucocorticoid-Induced Tumor Necrosis Factor Receptor

**DOI:** 10.1371/journal.pone.0104669

**Published:** 2014-08-08

**Authors:** Mikiya Ishihara, Naohiro Seo, Jun Mitsui, Daisuke Muraoka, Maki Tanaka, Junichi Mineno, Hiroaki Ikeda, Hiroshi Shiku

**Affiliations:** 1 Department of Immuno-Gene Therapy, Mie University Graduate School of Medicine, Mie, Japan; 2 Department of Gastroenterological Surgery II, Hokkaido University Graduate School of Medicine, Hokkaido, Japan; 3 Gene Medicine Business Unit, Takara Bio Inc., Shiga, Japan; University of Pennsylvania School of Veterinary Medicine, United States of America

## Abstract

Oncolytic virotherapy combined with immunomodulators is a novel noninvasive strategy for cancer treatment. In this study, we examined the tumoricidal effects of oncolytic HF10, a naturally occurring mutant of herpes simplex virus type-1, combined with an agonistic DTA-1 monoclonal antibody specific for the glucocorticoid-induced tumor necrosis factor receptor. Two murine tumor models were used to evaluate the therapeutic efficacies of HF10 virotherapy combined with DTA-1. The kinetics and immunological mechanisms of DTA-1 in HF10 infection were examined using flow cytometry and immunohistochemistry. Intratumoral administration of HF10 in combination with DTA-1 at a low dose resulted in a more vigorous attenuation of growth of the untreated contralateral as well as the treated tumors than treatment with either HF10 or DTA-1 alone. An accumulation of CD8^+^ T cells, including tumor- and herpes simplex virus type-1-specific populations, and a decrease in the number of CD4^+^ Foxp3^+^ T regulatory cells were seen in both HF10- and DTA-1-treated tumors. Studies using Fc-digested DTA-1 and Fcγ receptor knockout mice demonstrated the direct participation of DTA-1 in regulatory T cell depletion by antibody-dependent cellular cytotoxicity primarily via macrophages. These results indicated the potential therapeutic efficacy of a glucocorticoid-induced tumor necrosis factor receptor-specific monoclonal antibody in oncolytic virotherapy at local tumor sites.

## Introduction

Oncolytic virotherapy has existed for over 100 years and is a promising method for the treatment of cancer patients because of the strong cytolytic response of virus-infected tumor cells; however, complications may result from the use of oncolytic viruses including toxicity against normal cells [1]–[3]. Thus, artificially modified oncolytic viruses have been engineered to achieve low toxicity against normal tissues together with sufficient antitumor activity. Oncolytic viruses that have been modified to express human cytokines, such as granulocyte macrophage colony-stimulating factor (GM-CSF) have the potential for future therapeutic use in the treatment of solid tumors. JX-594 is a GM-CSF-armed oncolytic poxvirus that has shown promising outcomes when administered by either intratumoral (i.t.) injection or intravenous (i.v.) infusion [4]–[8]. OncoVEX^GM-CSF^ is an oncolytic virus based on the JS-1 strain of herpes simplex virus type-1 (HSV-1) that has been engineered to express human GM-CSF [9]–[12]. The results of a phase III trial demonstrate that melanoma patients treated with this virus show statistically significant improvement with durable responses [12].

HSV infection in wide ranges of cell populations results in degenerative change and death [13]. HF10 is a spontaneous mutant of HSV-1 strain HF [14] that lacks neuroinvasiveness and is at least 10,000-fold less virulent than wild-type HSV-1 in mice [15]. In several clinical studies of cancer patients, HF10 has been shown to have antitumor effects [16]–[19]. In murine studies, HF10 packaged with a GM-CSF-expressing amplicon has been reported to exhibit more tumoricidal activity than intact HF10 [20], [21], supporting the hypothesis that HF10 exhibits maximal antitumor activity when used in combination with immunomodulators.

Glucocorticoid-induced tumor necrosis factor receptor (GITR) is a type I transmembrane protein of the tumor necrosis factor receptor family, and is involved in the regulation of T-cell receptor-mediated cell death [22]. GITR is similar to programmed cell death-1 (PD-1) and cytotoxic T-lymphocyte antigen 4 (CTLA-4), both of which have been applied clinically as immune modifiers in tumor therapy [23]. GITR has been reported to be expressed at high levels on CD4^+^ CD25^+^ regulatory T (Treg) cells and to abrogate Treg cell-mediated immune suppression via intercellular signaling [24], [25]. GITR has also been known to be expressed on activated CD8^+^ T cells and to act on the induction of tumor-specific CD8^+^ T cells [26]. In addition, GITR signaling via specific ligands seems to drive CD8^+^ T cell resistance to Treg cell-mediated inhibition [27]. Currently there is an ongoing clinical trial of a therapeutic anti-human GITR antibody [28]. Thus, GITR targeting is an attractive candidate method for use in HF10 virotherapy as it encourages tumoricidal cytotoxic T lymphocyte (CTL) activity and attenuates immune suppression.

In this study, we examined the anti-tumor effects of i.t. treatment of established murine tumors with HF10 in combination with the GITR-specific agonistic monoclonal antibody (mAb) DTA-1. Our results show that the combination therapy inhibited tumor growth at the contralateral as well as the injected tumor sites by promoting the accumulation of tumor-specific CD8^+^ T cells followed by DTA-1-mediated depletion of CD4^+^ Foxp3^+^ Treg cells. Thus, DTA-1 is an extremely effective partner for HF10 in oncolytic virotherapy.

## Materials and Methods

### Mice

Female BALB/c mice aged 6–8 weeks were obtained from SLC Japan. BALB/c mice deficient in the γ subunit of the FcγRI, FcγRIII and FcεRI receptors (FcRγ KO mice) [29] were purchased from Taconic and bred at the Mie University Institute of Laboratory Animals. Experimental protocols were approved by the Animal Ethics Committee of Mie University, Tsu, Japan (Approval number: 23-8).

### Cell lines

CT26 is a colon tumor cell line derived from BALB/c mice [30]. A CT26 cell line transfected with the gene encoding the human cancer/testis antigen NY-ESO-1 (CT26/NY-ESO-1) was established as described previously [31]. CMS5a is a 3-methyl cholanthrene-induced fibrosarcoma cell line derived from BALB/c mice [32]. A CMS5a cell line transfected with the gene encoding GITR was established by retrovirus-mediated gene transfer. The retrovirus containing the murine GITR gene was purchased from Takara Bio Inc.

CT26/NY-ESO-1 and CMS5a cells were inoculated subcutaneously (s.c.) into the hind flanks of mice (1×10^6^ cells/mouse and 2×10^5^ cells/mouse, respectively). HF10 or the vehicle was administered i.t. (1×10^7^ PFU/mouse) at 7, 8, and 9 days after tumor inoculation. DTA-1 was administered i.t. (10 µg/mouse) at 9 days after tumor inoculation. For the combination therapy, 10 µg of DTA-1 were mixed with the HF10 virus and administered to the mice at 9 days after tumor inoculation.

### Antibodies

Fluorescein isothiocyanate (FITC)-conjugated and/or phycoerythrin (PE)-conjugated anti-mouse CD4 (RM4-5; eBioscience, Inc), anti-mouse CD8α mAb (53-6.7; BD Bioscience), anti-mouse/rat Foxp3 mAb (FJK-16s; eBioscience, Inc), anti-mouse IFN-γ mAb (XMG1.2; eBioscience, Inc), anti-mouse F4/80 mAb (BM8; BioLegend), and anti-rat IgG2b monoclonal antibodies (mAbs) (MRG2b-85; BioLegend) as well as a FITC-conjugated rabbit anti-HSV-1 polyclonal antibody (Dako) were used in flow cytometric analysis and immunohistochemstry. An anti-mouse CD16/CD32 mAb (93; eBioscience, Inc) was used for Fc-blocking in all experiments. For *in vivo* administration, anti-mouse GITR mAb (DTA-1, rat IgG2b) and anti-mouse CD8α mAb were purified by protein G affinity column chromatography of ascites from BALB/c nude mice intraperitoneally inoculated with a 53-6.7 hybridoma. Purifed rat serum IgG (Sigma) was used as the control antibody for all experiments with DTA-1. The Fab portion of DTA-1 was prepared by using a Pierce Fab Preparation Kit (Thermo Fisher Scientific) according to the manufacturer's instructions.

### Cell preparations

To collect tumor-infiltrating lymphocytes (TILs), a gentleMACS dissociator (Miltenyi Biotec K.K.) was used according to the manufacturer's instructions with some modifications. Briefly, a CT26/NY-ESO-1 tumor cut into small pieces was incubated in 4.5 mL of RPMI-1640 medium supplemented with 1 mg/mL collagenase IA (Sigma) for 40 min at 37°C and then dissociated into single cells using the gentleMACS dissociator. DNase I was not used. The obtained cells were passed through a cell strainer (70 µm) to remove tissue aggregates. After washing 3 times with PBS containing 0.1% BSA, the TILs were evaluated by flow cytometric analysis for intracellular IFN-γ as described below. When DTA-1-binding TIL populations were studied, collagenase I was not used so as to avoid the dissociation of DTA-1 bound to cells.

To investigate DTA-1-mediated generation of tumor-specific CD8^+^ T cells, DTA-1 was injected i.t. into day 9 CT26/NY-ESO-1 tumors at three doses (0.5, 2, or 10 µg). Tumor-regressed mice were selected from each DTA-1-treated group at 2 weeks after DTA-1 treatment, and the splenocytes from each group were pooled and incubated in RPMI-1640 medium supplemented with 10% fetal calf serum (FCS) and 10 µg/mL of control peptide (9 m: QYIHSANVL) [32], CT26-specific AH-1 peptide (SPSYVYHQF) [33] or NY-ESO-1_81–88_ peptide (RGPESRLL) [34] (all from MBL) for 5 hrs. The obtained cells were analyzed by flow cytometry to determine levels of intracellular IFN-γ.

Splenocytes from CT26/NY-ESO-1-bearing mice obtained at 5 days after i.t. treatment with both HF10 (days 7, 8 and 9) and DTA-1 (day 9) were cultured with CT26-specific AH-1 peptide (10 µg/mL) or HF10-infected CMS5a tumor cells [precultured with HF10 (MOI 1) for 12 hrs and irradiated with 50 Gy] at a ratio of 5 splenocytes to 1 HF10-infected CMS5a cell for 5 hrs. The obtained cells were then evaluated for intracellular IFN-γ as described below.

### Flow cytometric analysis of mAb-stained cells

To confirm GITR expression on CMS5a/GITR cells, CMS5a/GITR cells were incubated with DTA-1 (<2×10^6^ cells/µg in PBS supplemented with 0.2% BSA) for 15 min at 4°C. After washing 3 times with PBS containing 0.1% BSA, the cells were further treated with FITC-conjugated anti-rat IgG (H + L) Ab (Caltag Lab.) (<2×10^6^ cells/µg) for 15 min at 4°C. For staining of intracellular IFN-γ in cultured splenic CD8^+^ T cells, GolgiPlug (BD Bioscience) protein transport inhibitor was added for the last 4 hrs of the incubation. The obtained cells were permeabilized using a Cytofix/Cytoperm Kit (BD Bioscience) and stained with a CD8α-specific mAb for 15 min at 4°C and followed by an IFN-γ-specific mAb for 15 min at 4°C (<2×10^6^ cells/µg). For Foxp3-CD4 double labeling of TILs, TILs were first stained with a CD4-specific mAb [15 min, 4°C (<2×10^6^ cells/µg)], then fixed and permeabilized using a Foxp3 staining kit (eBioscience, Inc.), and then treated with a Foxp3-specific mAb [30 min, 4°C (<1×10^6^ cells/µg)]. The labeled cells were then analyzed by flow cytometry (FACSCanto II: BD Bioscience) with FlowJo software (Tomy Digital Biology).

### Immunohistochemistry

Frozen CT26/NY-ESO-1 tumor specimens embedded in O.C.T compound (Sakura Finetechnical) were sectioned at a thickness of 3 µm, air dried for 2 hrs, fixed with cold acetone for 15 min, and then processed for immunohistochemistry. After washing 3 times with PBS, the slides were incubated at 4°C in blocking solution [PBS supplemented with 1% BSA, 5% Blocking One Histo (Nacalai Tesque, Inc.)] and 0.2 µg/mL anti-mouse CD16/CD32 mAb for 30 min. The tumor sections on the slides were then dual-labeled with PE-conjugated mAb and FITC-conjugated mAb diluted with PBS supplemented with 1% BSA and 5% Blocking One Histo for 1 hr at room temperature (r.t.) in a humidified chamber. After washing 3 times with PBS supplemented with 0.02% Tween-20, the slides were mounted in ProLong Gold antifade reagent with DAPI (Invitrogen, Life Technologies, Inc.), and evaluated by fluorescence microscopy (BX53F; Olympus Co. Ltd.; Tokyo, Japan). The photographs from PE-, FITC-, and DAPI-stained tissue sections were merged and background fluorescence was deleted using Photoshop elements software (Adobe Systems Software Ltd.).

For hematoxylin and eosin (HE) staining, slides with acetone-fixed tissue sections were washed 3 times with PBS and incubated at r.t. in hematoxylin solution (New Hematoxylin Sol.: Muto Pure Chemicals Co., Ltd.) for 5 min. After washing with tap water, the cytoplasm was stained with eosin (r.t. for 2 min.; Pure Eosin Sol.: Muto Pure Chemicals). Samples were then dehydrated 3 times with xylene, and the slides were mounted with Malinol (Muto Pure Chemicals) and evaluated by microscopy.

### Antibody-dependent cellular cytotoxicity (ADCC) assay

RAW264.7 cells were activated with 20 ng/mL murine IFN-γ for 24 hrs in 24-well plates, after which the cells were gently washed with RPMI-1640 and the dish-adherent RAW264.7 cells were used as effectors in the ADCC assay. CMS5a or CMS5a/GITR cells were labeled with 2.5 µM carboxyfluoresceine diacetate succinimidyl ester (CFSE) at 37°C for 6 min to be used as targets in the ADCC assay. After washing 3 times with RPMI-1640 supplemented with 10% FCS, CFSE-labeled CMS5a or CMS5a/GITR cells were plated at various effector-to-target ratios with rat IgG or DTA-1 (2 µg/mL), incubated for 12 hrs, and analyzed by flow cytometry. For each sample, 20,000 non-CFSE labeled cells were collected, and the absolute number of CFSE-labeled surviving cells was counted. The survival percentage was calculated as the mean number of each of the three wells as follows: [(absolute number of surviving CFSE-labeled cells in control rat IgG-containing medium)−(absolute number of surviving CFSE-labeled cells in DTA-1-containing medium)]×100/(absolute number of surviving CFSE-labeled cells in control rat IgG-containing medium).

### Statistical analysis

The Mann-Whitney U test was used to compare data from two groups. When equality of variance was proven by Levine's test, data comparison between 2 groups was evaluated by Student's *t*-test. The Kruskal-Wallis ANOVA test was used to compare data from four groups. *p*-values below 0.05 were considered statistically significant. Calculations were performed using SPSS Statistics v21.0 software (IBM).

## Results

### Effective inhibition of tumor growth by local treatment of HF10 combined with DTA-1

**Figure 1:**
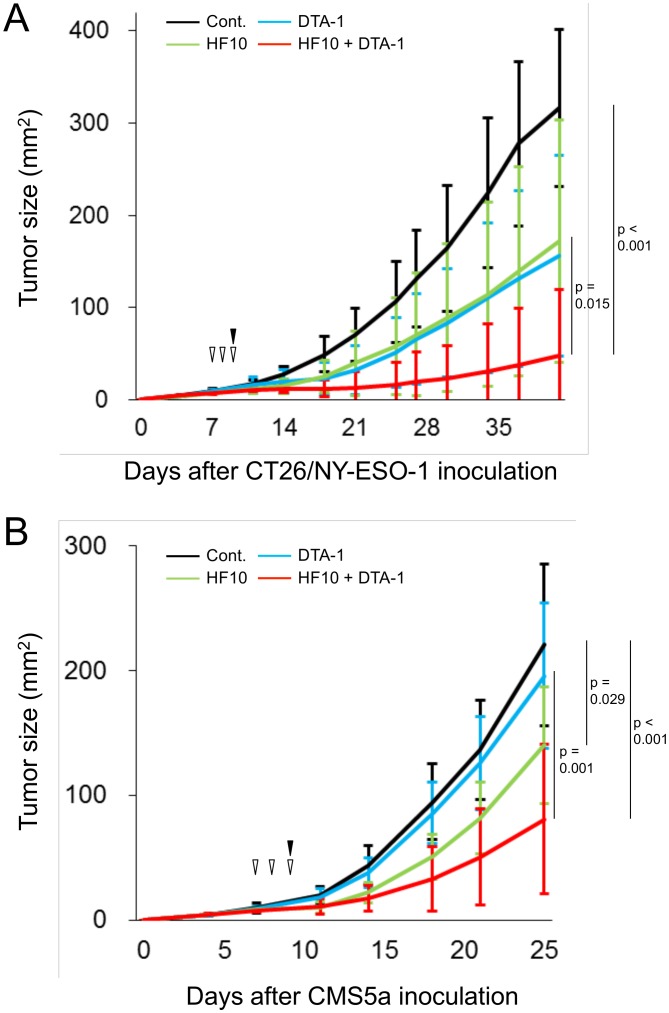
Tumor growth following intratumoral administration of HF10 with/without DTA-1. Growth (mm^2^) of subcutaneously inoculated CT26/NY-ESO-1 (A) or CMS5a cells (B) following i.t. treatment with HF10 (open triangles) and/or DTA-1 (closed triangles) on indicated days. Purified rat IgG was used as a control for DTA-1. Data shown in Fig. 1 1 are representative of four independent experiments. By the Kruskal-Wallis ANOVA test, the CT26/NY-ESO-1 growth inhibition by combined treatment with HF10 and DTA-1 was significantly different from the HF10-treated or untreated control at day 42. CMS5a growth inhibition by the HF10 and DTA-1 combination was significantly different from the DTA-1-treated or untreated control at day 25. CMS5a growth inhibition was also significantly different in the HF10-treated group compared with the untreated control.

Since the therapeutic efficacy of the adjuvants included in immune-targeting Abs has been widely shown in the treatment of cancer, we hypothesized that the use of DTA-1, as an enhancer of tumor-specific CD8^+^ T cell responses [26], [27] in HF10 virotherapy might produce a satisfactory treatment outcome. To investigate this hypothesis, we used human NY-ESO-1 gene-transfected CT26 tumor cells (CT26/NY-ESO-1) for *in vitro* and *in vivo* studies as an H-2D^d^-restricted murine CTL epitope of NY-ESO-1 had been identified in our laboratory [31]. Subcutaneously inoculated CT26/NY-ESO-1 tumors were treated by i.t. administration of HF10 with or without DTA-1 (Fig. 1A). Groups treated with either HF10 or DTA-1 showed weak suppression of tumor growth compared with the untreated group (control), and 2 out of 13 mice (15.4%) or 3 out of 19 mice (15.8%) showed complete tumor regression at 42 days after tumor inoculation, respectively (Table 1). In contrast, all mice in the group treated with both HF10 and DTA-1 showed statistically significant attenuation of tumor growth compared with the control group [*p*<0.001, Fig. 1A; complete tumor regression rate at 42 days: 60.0% (12 to 20 mice); Table 1]. In addition, CMS5a tumor growth in the group treated with both HF10 and DTA-1 was also suppressed significantly compared with the control or DTA-1-treated groups (Fig. 1B and Table 1).

**Table 1:** Increase in the number of tumor-regressed mice by HF10 therapy combined with DTA-1.

Treatment	Number of complete tumor-regressed mice*/Number of treated mice (%)	
	CT26/NY-ESO-1	CMS5a
Cont. IgG	0/10 (0.0%)	0/13 (0.0%)
DTA-1	2/13 (15.4%)	0/13 (0.0%)
HF10	3/19 (15.8%)	0/13 (0.0%)
HF10+DTA-1	12/20 (60.0%)	3/13 (23.1%)

* Number of complete tumor-regressed mice was counted at 42 or 25 days after subcutaneous inoculation of CT26/NY-ESO-1 or CMS5a tumor cells, respectively.

We observed CT26/NY-ESO-1-regressed mice in another experiment for 2 months after HF10 and DTA-1 treatment. Tumor recurrence could not be seen in the tumor-regressed mice. In addition, these mice exhibited the resistance in tumor re-challenging.

### CD8^+^ T cells act as tumoricidal effectors in the combination therapy of HF10 with DTA-1

**Figure 2:**
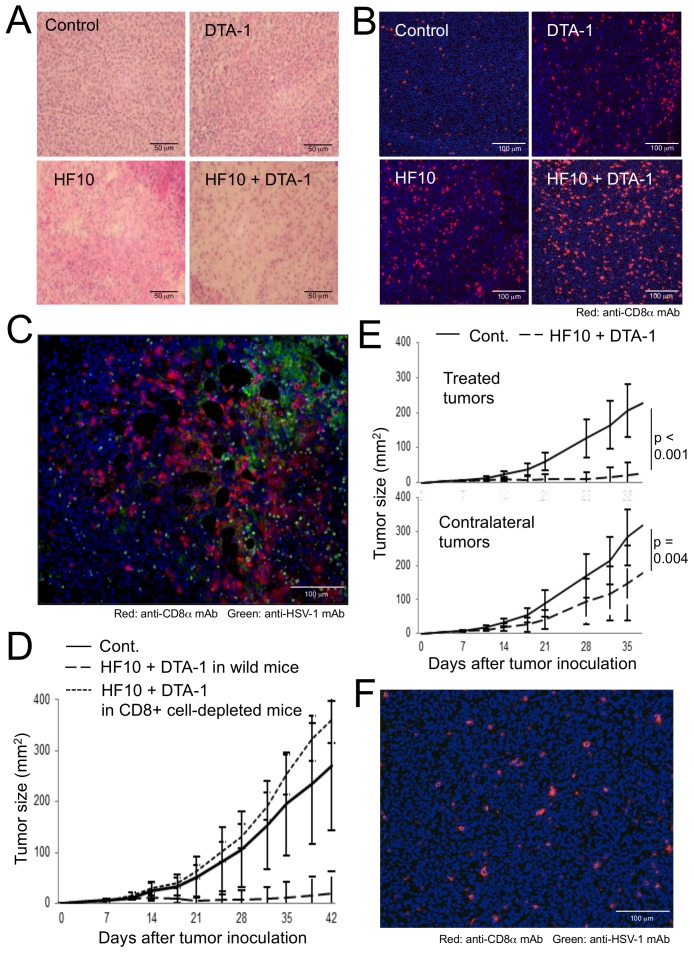
Kinetics of CD8^+^ T cells after the combination therapy with HF10 and DTA-1. CT26/NY-ESO-1 tumor sections from untreated mice (control) or mice injected i.t. with DTA-1, HF10, or HF10 combined with DTA-1 were stained with hematoxylin and eosin (A), and phycoerythrin (PE)-conjugated anti-CD8α mAb and DAPI (B). (C) Frozen sections of CT26/NY-ESO-1 tumors from mice i.t. injected with HF10 combined with DTA-1 were stained with a PE-anti-CD8α monoclonal antibody, a fluorescein isothiocyanate (FITC)-anti-HSV-1 polyclonal antibody, and DAPI. (D) CT26/NY-ESO-1 growth (mm^2^) in both i.t. HF10- and DTA-1-treated control or CD8^+^ cell-depleted mice was measured. Seven mice per group were used. (E) Bilateral CT26/NY-ESO-1-bearing mice were treated with a combination of HF10 and DTA-1 in the tumors on the right flanks. Subsequent tumor growth (mm^2^) of the treated right and contralateral left sites was measured. Tumor growth in untreated mice was measured and used as a control. Fourteen mice per group were used. By the Kruskal-Wallis ANOVA test, CT26/NY-ESO-1 growth inhibition by the combined HF10 and DTA-1 treatment in contralateral as well as treated sites was significantly different from the untreated control group. (F) CT26/NY-ESO-1 tumors from one side of bilateral tumor-bearing mice were treated i.t. with HF10 combined with DTA-1. Frozen sections of contralateral CT26/NY-ESO-1 tumors were stained with a PE-anti-CD8α monoclonal antibody, a fluorescein isothiocyanate (FITC)-anti-HSV-1 polyclonal antibody, and DAPI.

Intratumoral injection of HF10 resulted in the collapse of tumor structure with a decrease in the nuclear density of tumor cells, as shown at 7 days after the last HF10 treatment in both the group treated with HF10 and that treated with HF10 and DTA-1 (Fig. 2A). Tumor-infiltrating CD8^+^ T cells were shown to be the most frequent population after the administration of HF10 combined with DTA-1 at 3 days after the final treatment (Fig. 2B). Importantly, these cells appeared to accumulate near HF10-infected tumor areas (Fig. 2C and S1A), suggesting that HF10 infection is able to attract CD8^+^ T cells by leaking virus-associated proteins and tumor antigenic proteins from infected tumor cells and changing the tumor microenvironment after oncolysis. Inhibition of CT26/NY-ESO-1 growth by the combination therapy was completely negated by depleting CD8^+^ cells by intravenous treatment with a murine CD8α-specific mAb (Fig. 2D), indicating that the tumor-infiltrating CD8^+^ T cells shown in Figure 2B and 2C include tumoricidal effector populations. In the study using bilateral tumor-bearing mice, tumor growth inhibition by HF10 combined with DTA-1 occurred not only in the treated tumors but also in the contralateral non-treated tumors (Fig. 2E and S1B). In addition, the sections of contralateral tumor showed infiltrating CD8^+^ T cells without HF10 infection (Fig. 2F and S1C). These results indicate that CD8^+^ T cells activated in a local tumor site under the influence of HF10 and DTA-1 participate in systemic surveillance and could attack distant tumors without tissue destruction due to HF10 infection.

### Augmentation of tumor-specific CD8^+^ T cell responses by DTA-1 treatment in HF10 therapy

**Figure 3:**
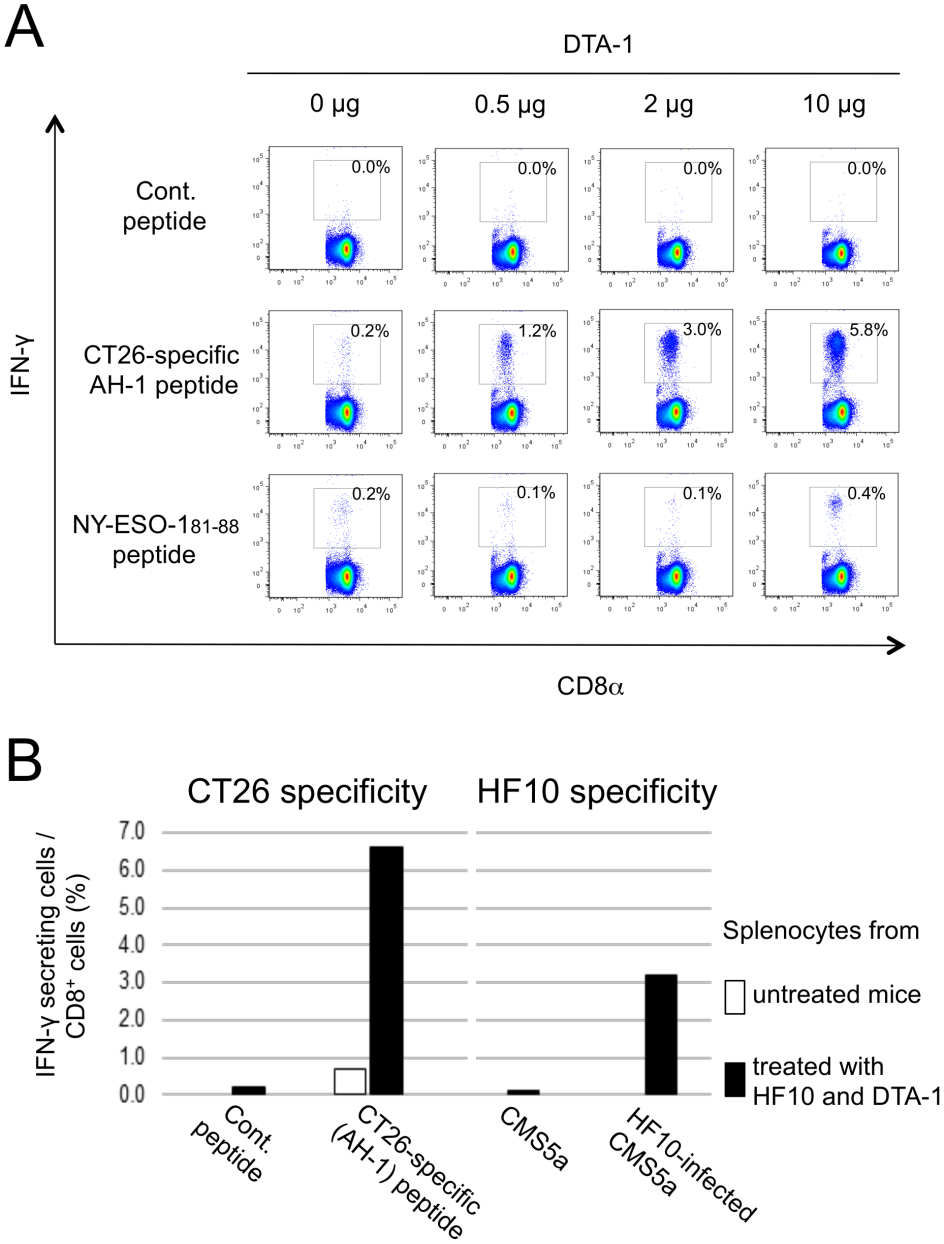
Generation of tumor- and HF10-specific CD8^+^ T cells by intratumoral treatment of DTA-1 and HF10 combined with DTA-1. (A) CT26- and NY-ESO-1-specific CD8^+^ T cell responses in CT26/NY-ESO-1-regressed mice by i.t. treatment of DTA-1 at indicated doses were assessed by intracellular staining of IFN-γ in splenocytes cultured with 10 µg/mL of the indicated peptides for 5 hrs. Splenocytes from two mice per group were pooled and assessed. hrs. Splenocytes from two mice per group were pooled and assessed. (B) Splenocytes from untreated and both i.t. HF10- and DTA-1-treated CT26/NY-ESO-1-bearing mice were obtained at 5 days after final treatment, and cultured with AH-1 peptide (10 µg/mL) or HF10-infected CMS5a tumor cells for 5 hrs. Splenocytes from ten mice per group were pooled and assessed. The obtained cells were immunohistologically stained for intracellular IFN hrs. Splenocytes from ten mice per group were pooled and assessed. The obtained cells were immunohistologically stained for intracellular IFN-γ. The 9 m peptide and uninfected CMS5a cells were used as controls. m peptide and uninfected CMS5a cells were used as controls.

Next, we investigated whether the tumor- or HF10-specific CD8^+^ T cell response was enhanced in CT26/NY-ESO-1-bearing mice by i.t. treatment with DTA-1 alone or HF10 combined with DTA-1. To detect low proportions of CD8^+^ T cells with tumor specificity, spleen cells from tumor-regressed mice selected after i.t. treatment with DTA-1 at the indicated doses were stimulated with a CT26-specific AH-1 peptide or an NY-ESO-1 81–88 peptide to expand each population of peptide-specific CD8^+^ T cells. As shown in Figure 3A, the response of CT26-specific IFN-γ-producing CD8^+^ T cells was enhanced by DTA-1 in a dose-dependent manner. In addition, CD8^+^ T cells with NY-ESO-1 specificity were observed when DTA-1 was administered at a high dose (10 µg). In this experiment, we used tumor-regressed mice because we could not enhance the negligible CT26-specific IFN-γ-producing CD8^+^ T cell responses seen in tumor-bearers in a DTA-1 dose-dependent manner. Although CT26/NY-ESO-1 growth after i.t. treatment with DTA-1 was suppressed compared with the control group, tumor size was not different in each group of DTA-1 (0.5, 2.0, or 10.0 µg/mouse). Furthermore, HF10-specific CD8^+^ T cells were found in addition to the AH-1-specific population (Fig. 3B left) when splenocytes from both HF10- and DTA-1-treated CT26/NY-ESO-1-bearing mice with HF10-infected CMS5a cells were cultured (Fig. 3B right). These results indicated that i.t. treatment of HF10 and DTA-1 had the capacity to enhance tumor- and HF10-specific CD8^+^ T cell populations.

### Disappearance of tumor-infiltrating Foxp3^+^ cells after the treatment with DTA-1 in HF10 therapy

**Figure 4:**
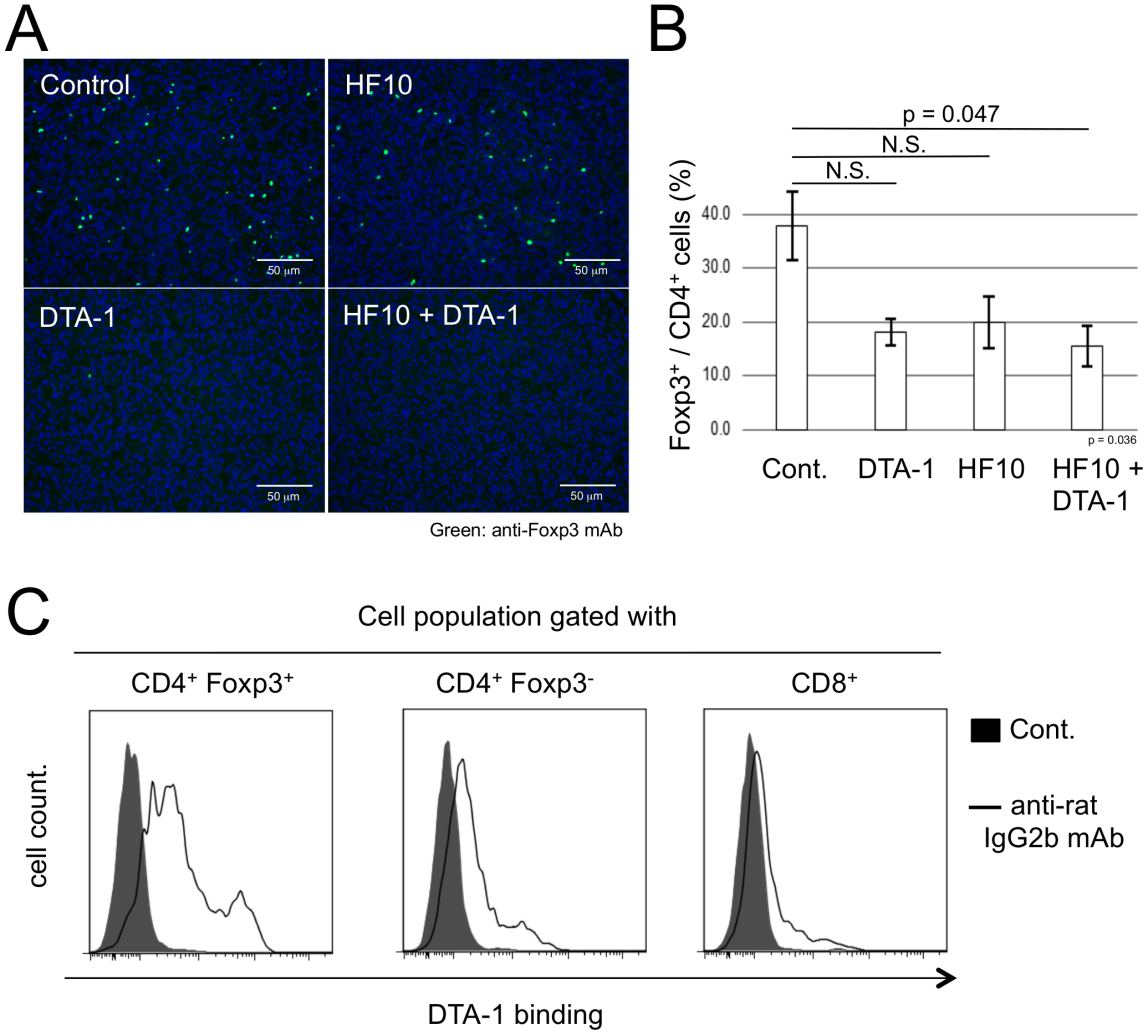
Disappearance of DTA-1-conjugated tumor-infiltrating CD4^+^ Foxp3^+^ cells after combined i.t. treatment with HF10 and DTA-1. (A) CT26/NY-ESO-1 tumor sections form untreated group (control) or group from mice injected i.t. with DTA-1, HF10, or HF10 combined with DTA-1 were stained with FITC-anti-Foxp3 mAb and DAPI. (B) The frequency of tumor-infiltrating CD4^+^ Foxp3^+^ Treg cells from mice injected i.t. with HF10, DTA-1, or HF10 combined with DTA-1 at 12 days after CT26/NY-ESO-1 inoculation was assessed by flow cytometry. Data from 4 individual experiments were analyzed statistically. Kruskal-Wallis ANOVA test was used to compare data from the 4 groups. The decrease in the frequency of tumoral CD4^+^ Foxp3^+^ Treg cells in the HF10 and DTA-1 combined treatment group was significantly different from untreated control, but not from the HF10- or DTA-1-treated group (N.S: Not significant). (C) At 6 hrs after DTA hrs after DTA-1 injection into day 9 CT26/NY-ESO-1 tumors, tumor-infiltrating cells collected under collagenase-free conditions were analyzed by flow cytometry after staining with FITC-labeled anti-rat IgG2b mAb to detect DTA-1-bound cells.

We hypothesized that the increase in tumor-specific CD8^+^ T cell responses after DTA-1 treatment combined with HF10 therapy was involved in the attenuation and/or depletion of immune suppressors including Treg cells. To address this issue, CT26/NY-ESO-1 tumors obtained after DTA-1 treatment with or without HF10 were evaluated by immunohistological staining of tissue sections and flow cytometric analysis of infiltrating lymphocytes using a Foxp3-specific mAb. Foxp3^+^ cells accumulated abundantly in both untreated and HF10-treated tumors, whereas a vigorous decrease in the number of Treg cells was shown in tumors following treatment with DTA-1 alone or DTA-1 combined with HF10 (Fig. 4A). This result was confirmed by flow cytometric analysis of tumor-infiltrating Treg cells. The frequency of tumoral CD4^+^ Foxp3^+^ Treg cells from the HF10- and DTA-1-treated group was decreased significantly compared with that from the untreated (control) group but not from the HF10- or DTA-1- treated mice (Fig. 4B). The decrease in the frequency of Foxp3^+^ cells in the HF10-treated group (Fig. 4B) is possibly correlated with the decrease in tumor size due to HF10 treatment. This may be attributed to the lack of modulation of the absolute number of Foxp3^+^ cells in the HF10-treated group unlike in the control group (Fig. 4A). DTA-1 is a rat IgG2b class mAb. By visualizing DTA-1 with the FITC-conjugated anti-rat IgG2b mAb, it was demonstrated that the tumor-infiltrating CD4^+^ Foxp3^+^ Treg population bound predominantly with DTA-1 at 6 hrs after i.t. injection (Fig. 4C), in parallel with the disappearance of tumoral Treg cells after treatment with DTA-1. Taken together, these results strongly indicate that DTA-1 was essential to the decrease in the number of CD4^+^ Foxp3^+^ cells.

### Depletion of tumor-infiltrating Treg cells by DTA-1-mediated cellular cytotoxicity

**Figure 5:**
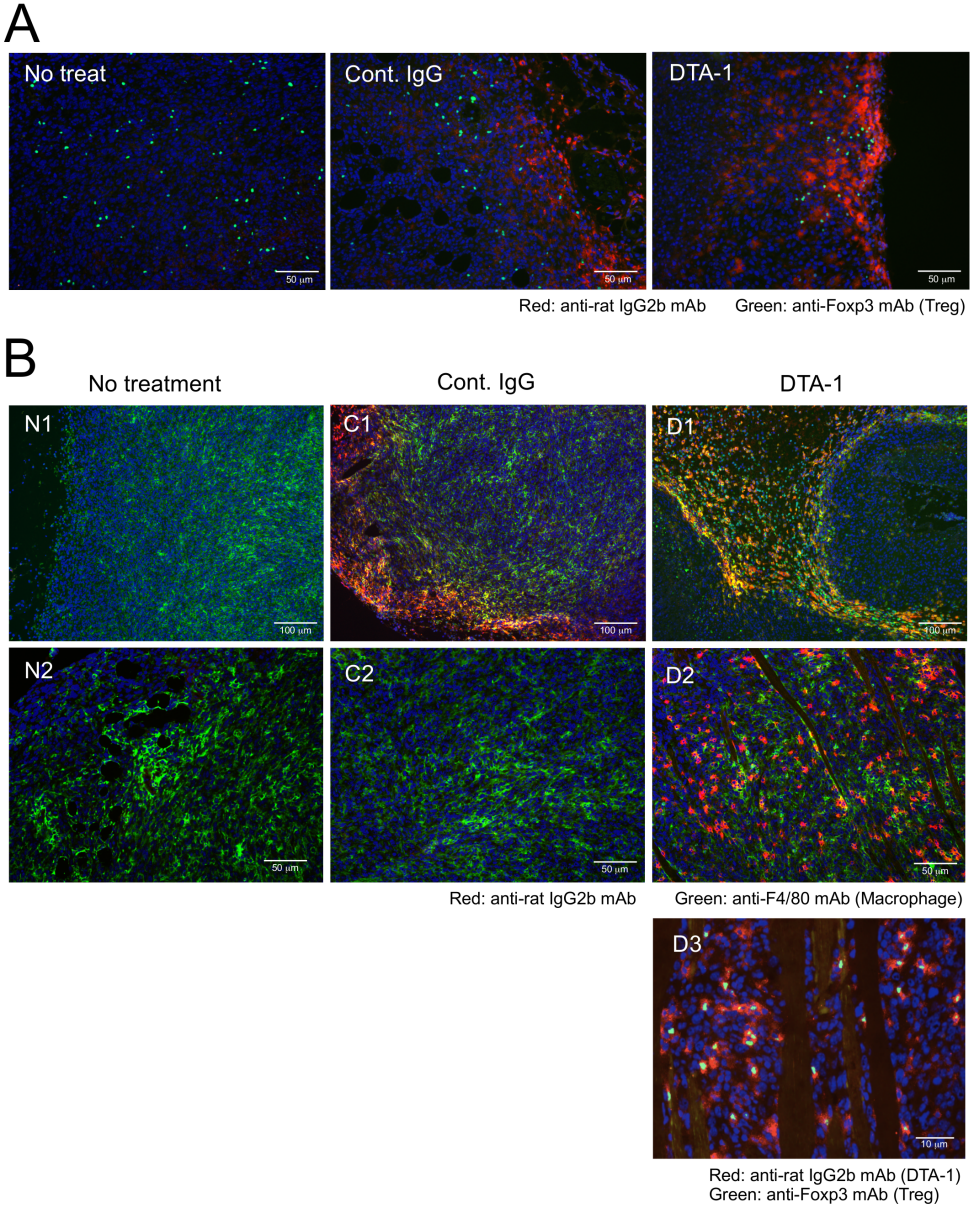
Kinetics of Foxp3^+^ Treg cells and F4/80^+^ macrophages in DTA-1-treated tumors. Frozen sections of CT26/NY-ESO-1 tumors obtained at 6 hrs after DTA hrs after DTA-1 i.t. injection were stained with FITC-conjugated anti-Foxp3 mAb, PE-conjugated anti-rat IgG2b mAbs and DAPI (A and B: D3), or FITC-anti-F4/80 mAb, PE-anti-rat IgG2b mAb, and DAPI (B: D1 and D2). Sections from untreated and control rat IgG-treated tumors were used as controls (A and B: N1, N2, C1, C2). Representative photos from three experiments are shown.

Fluorescent immunohistological studies using double labeling with anti-rat IgG2b mAb (for DTA-1) and F4/80- (for macrophages) or Foxp3- (for Tregs) specific mAbs were performed to determine the mechanisms of DTA-1-dependent depletion of CT26/NY-ESO-1 tumor-infiltrating Treg cells. At 6 hrs after DTA-1 treatment, Foxp3^+^ cells clustered at the DTA-1-stained peritumor sites, whereas Foxp3^+^ cells did not accumulate in the control rat IgG-treated case (Fig. 5A and S2A). Images of red fluorescence from DTA-1 or the control rat IgG merged with the green fluorescence from F4/80^+^ macrophages in nearby tumor stroma (Fig. 5B; C1, D1, and S2B) indicated that DTA-1 and rat IgG bound with macrophage-expressing FcRs. In addition, a large number of cells visualized in lymphocyte-like formation by staining with anti-rat IgG2b mAb were positive for Foxp3 (Fig. 5B; D2, D3; Fig. S3A and B) and were in contact with macrophages in various areas of DTA-1–treated tumors (Fig. 5B; D2; Fig. S3A). These results strongly support the hypothesis that DTA-1 participates in GITR^+^ Foxp3^+^ Treg depletion by ADCC at the treated tumor sites.

**Figure 6:**
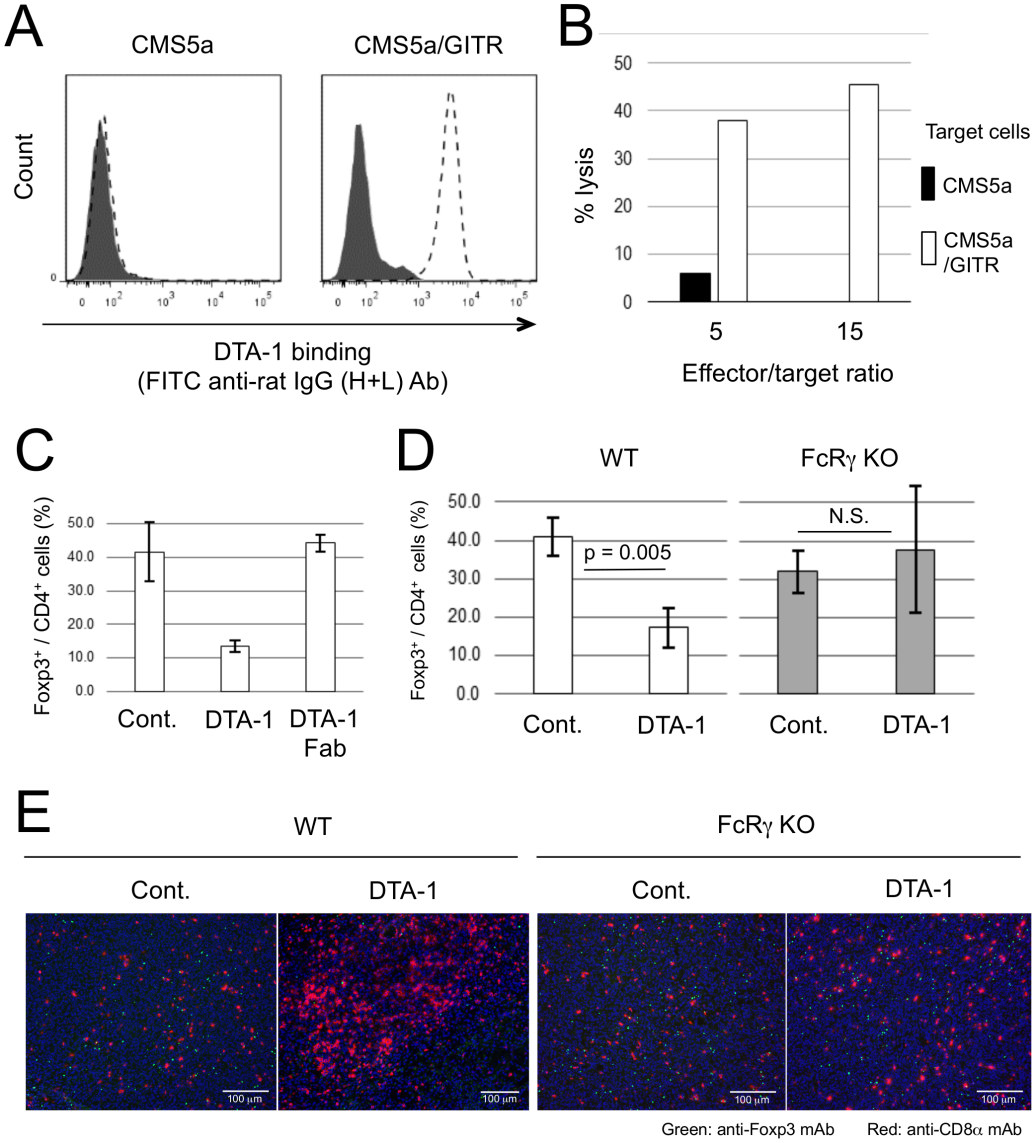
DTA-1-mediated depletion of tumor-infiltrating CD4^+^ Foxp3^+^ Treg cells by ADCC. (A) DTA-1- (dotted line) or isotype control (solid line)-treated CMS5a and murine GITR gene-transfected CMS5a (CMS5a/GITR) cells were stained with a FITC-conjugated anti-rat IgG (H+L) antibody and analyzed by flow cytometry. (B) CFSE-labeled CMS5a and CMS5a/GITR cells were used as targets. The mixture of IFN-γ-activated RAW264.7 cells (effector cells) and target cells were incubated for 12 hrs with control IgG or DTA hrs with control IgG or DTA-1 at effector/target ratios of 5 and 15. (C) Frequency of Foxp3^+^ cells in tumor-infiltrating CD4^+^ cell population at 3 days after i.t. DTA-1 or Fc-digested DTA-1 (DTA-1 Fab) treatment was measured by flow cytometric analysis. (D) Frequency of Foxp3^+^ cells in tumor-infiltrating CD4^+^ cell population at 3 days after DTA-1 i.t. treatment in wild-type or FcRγ KO mice was measured by flow cytometric analysis. By Student's t-test, the decrease in the frequency of Foxp3^+^ cells in DTA-1-treated CT26/NY-ESO-1 tumors of wild type mice, but not FcRγ KO mice (N.S.: Not significant), was significantly different from untreated control group. (E) Frozen sections of CT26/NY-ESO-1 tumors obtained at 3 days after DTA-1 i.t. treatment in wild type and FcRγ KO mice were stained with FITC-anti-Foxp3 and PE-anti-CD8α mAbs, and DAPI.

To examine whether DTA-1 can mediate ADCC in a murine system, we performed an *in vitro* ADCC assay using IFN-γ-activated RAW264.7 macrophage cells as an effector and murine GITR gene-transfected CMS5a (CMS5a/GITR) cells (Fig. 6A) as a target. CMS5a/GITR cells were lysed in the presence of DTA-1 in a GITR-specific manner (Fig. 6B). We further investigated *in vivo* the ADCC effects of DTA-1 using Fc portion-digested DTA-1 (DTA-1 Fab) and FcRγ KO mice. Depletion of CD4^+^ Foxp3^+^ Treg cells in CT26/NY-ESO-1 tumors was not observed following i.t. DTA-1 Fab treatment (Fig. 6C). In addition, no significant decreases in the number of CD4^+^ Foxp3^+^ Treg cells and accumulation of CD8^+^ T cells were detected by DTA-1 treatment in FcRγ KO mice, unlike the results from wild-type mice (Fig. 6D, 6E, and S4). These results clearly indicated the direct participation of DTA-1 in Treg cell depletion by ADCC.

Taken together, these results show that HF10 virotherapy combined with DTA-1 elicits a powerful therapeutic effect against tumors via the accumulation of CD8^+^ T cells, after tumor destruction by HF10 and the enhancement of tumor- and virus-specific CD8^+^ T cell responses directly or indirectly by depletion of immune-suppressive Treg cells at tumor sites by DTA-1.

## Discussion

Many studies involving oncolytic virus combined with systemic administration of cytotoxic agents have shown promising results in animal models. However, almost all of the studies have avoided the important issue of lymphocyte suppression caused by steroids as an antiemetic, implying the clinical inapplicability of such cytotoxic agents. Tumor therapy promises an era of safety in using noninvasive immunomodulatory agents including PD-1-, CTLA-4- and GITR-specific mAbs. Unfortunately, all of them have produced slight immune-related slight adverse events such as diarrhea, rashes or pruritis [35]–[38]. In addition, systemic administration of immunomodulators can elicit serious autoimmune diseases. A study from another group has shown that in a murine model, treatment with 50 µg/mouse DTA-1 induces antitumor activity and weak autoimmune reactions [39]. In this study, we also demonstrated that HF10 virotherapy combined with a GITR-targeting mAb in local tumor sites at more clinical appropriate lower and safer doses, elicits tumor lysis by augmented systemic tumor-specific CD8^+^ T cell activity with negligible toxicity. Therefore, local treatment of immunomodulators is a promising method for the future treatment of tumors.

The use of blocking Abs for suppressing immune signals has shown clinical benefits in the treatment of solid tumors [40]–[42]. Both PD-1 and CTLA-4, which are expressed on activated T cell surfaces, inhibit tumoricidal effector T cell responses by engagement via specific ligands that are expressed on various tumor cells [23]. However, high densities of tumor-infiltrating CD4^+^ CD25^+^ Foxp3^+^ Treg cells have been correlated with poor survival [43]–[45]. Treg cells express both PD-1 and CTLA-4 in the steady state without activation. PD-1 and CTLA-4 signals result in Treg induction and maintenance, and subsequent outbreak of autoimmune diseases [46]. Interestingly, it has been reported that an anti-CTLA-4 antibody augments tumoricidal effector T cells by downregulation of Treg cell functions, including ADCC-mediated depletion of Treg cells [47], which is similar to our GITR-targeting results. These reports indicate that the blockade of immune checkpoint molecules involves the activation of tumoricidal effector T cells by preventing interactions with specific ligands on tumor cells and inhibiting Treg cell functions.

The expression of GITR has been observed on CD4^+^ CD25^+^ Treg cells at relatively high levels [24], [25], which is consistent with our results. In addition, the GITR-GITRL interaction has been known to attenuate Treg cell function via the loss of Foxp3 expression as well as enhance tumor-specific effector CD4^+^ and CD8^+^ T cell functions [27], [39], [48], [49]. In this study, we demonstrated the use of DTA-1 as a depletion antibody because Fc-digested DTA-1 and intact DTA-1 in FcRγ KO mice did not participate in the downregulation of Foxp3 expression. After i.t. DTA-1 injection, macrophages appeared to attract DTA-1-conjugated Treg cells via their FcR and migrate to peritumor sites, as shown in Fig. 5, and the results suggest that the peritumoral stroma is a crucial place for ADCC triggering. Since CCL22 secreted by macrophages is known to be a chemoattractant for Treg cells [43], [50], such chemokines might participate in DTA-1-mediated Treg cell depletion. Further studies are necessary to elucidate the molecular mechanisms of tumoral ADCC.

As indicated in Fig. 4C, a small proportion of tumor-infiltrating CD8^+^ T cells bound with i.t. treated DTA-1. In addition, DTA-1 enhanced tumor-specific CD8^+^ T cell responses in a dose-dependent manner in tumor-regressed mice as shown in Fig. 3. These results suggest that DTA-1 acts as a direct activator of CD8^+^ T cells, although we could not rule out the possibility that tumor-specific CD8^+^ T cell responses were increased by DTA-1 dose-dependent depletion of immune suppressive Treg cells. Indeed, it has been reported that the function and activity of CTLs are augmented by the signals through GITR [26], [48], [51]. In this study, HF10-specific CD8^+^ T cells were detected after both HF10 and DTA-1 injections, concomitant with vigorous tumor-specific CTL responses. La et al. have reported that DTA-1 elicits immediately explosive HSV-1-specific CD8^+^ CTL and CD4^+^ Th responses in HSV-1-infected mice [52]. In addition, we found in this study that DTA-1 was detected in tumor-draining lymph nodes soon after i.t. injection (Fig. S5), suggesting the relationship between DTA-1 and quick generation of tumor-specific CTLs. Thus, it is likely that HF10-specific CTL responses induced by DTA-1 change the tumor microenvironment to facilitate the expansion of CTLs in tumor-draining lymph nodes.

In conclusion, local HF10 therapy combined with DTA-1 should be suitable for the treatment of cancer patients without crucial side effects. The benefits of the combined treatment regimen include the vigorous expansion of tumoricidal CTLs associated with the early HF10-specific CTL responses, inhibition of tumor formation by HF10 infection, direct expansion of CD8^+^ T cells by DTA-1, and negation of immune suppressive Treg cell activities by DTA-1-mediated ADCC and/or DTA-1 signaling.

## Supporting Information

Figure S1 **Systemic surveillance of tumoricidal CTLs after HF10 combination therapy with DTA-1 at local tumor sites.** (A) The images of red (PE), green (FITC), and blue (DAPI) fluorescence that were merged to produce Fig. 2C. (B) Bilateral CT26/NY-ESO-1-bearing mice were treated i.t. with a combination of HF10 and DTA-1 in tumors on the right flanks of mice. Tumor growth in the treated right and contralateral left sites was measured. Photos show representative mice at 25 days after CT26/NY-ESO-1 inoculation from the control and dual HF10- and DTA-1-treated groups. (C) The three images of red (PE), green (FITC), and blue (DAPI) fluorescence that were merged to produce Fig. 2F.
(TIF)

Figure S2 **The three fluorescence components of the merged images of** **Fig. 5A** **and** **Fig. 5B** **(N1, C1, and D1).** Three separate images of red (PE), green (FITC), and blue (DAPI) fluorescence that were merged to produce Fig. 5A (A) and N1, C1, and D1 of Fig. 5B (B).
(TIF)

Figure S3 **The three fluorescence components of the merged images of N2, C2, D2, and D3 in** **Fig. 5B** **.** The three separate images of red (PE), green (FITC), and blue (DAPI) fluorescence that were merged to produce N2, C2, D2, and D3 images in Fig. 5B.
(TIF)

Figure S4 **The three fluorescence components of the merged images in** **Fig. 6E** **.** The three separate images of red (PE), green (FITC), and blue (DAPI) fluorescence that were merged to produce Fig. 6E.
(TIF)

Figure S5 **Drafting of i.t. treated DTA-1 into tumor-draining lymph nodes.** Frozen sections of tumor-draining lymph nodes obtained at 6 hrs after intratumoral DTA-1 or DTA-1 Fab treatment were stained with a FITC-conjugated anti-rat IgG2b antibody, a phycoerythrin (PE)-conjugated anti-F4/80 antibody, and DAPI.
(TIF)

## References

[1] Vaha-Koskela MJ, Heikkila JE, Hinkkanen AE (2007) Oncolytic viruses in cancer therapy. Cancer Lett 254: 178–216. 17383089 10.1016/j.canlet.2007.02.002 PMC7126325

[2] Breitbach CJ, Reid T, Burke J, Bell JC, Kirn DH (2010) Navigating the clinical development landscape for oncolytic viruses and other cancer therapeutics: no shortcuts on the road to approval. Cytokine Growth Factor Rev 21: 85–89. 20472490 10.1016/j.cytogfr.2010.02.001

[3] Eager RM, Nemunaitis J (2011) Clinical development directions in oncolytic viral therapy. Cancer Gene Ther 18: 305–317. 21436867 10.1038/cgt.2011.7

[4] Kim JH, Oh JY, Park BH, Lee DE, Kim JS, et al (2006) Systemic armed oncolytic and immunologic therapy for cancer with JX-594, a targeted poxvirus expressing GM-CSF. Mol Ther 14: 361–370. 16905462 10.1016/j.ymthe.2006.05.008

[5] Park BH, Hwang T, Liu TC, Sze DY, Kim JS, et al (2008) Use of a targeted oncolytic poxvirus, JX-594, in patients with refractory primary or metastatic liver cancer: a phase I trial. Lancet Oncol 9: 533–542. 18495536 10.1016/S1470-2045(08)70107-4

[6] Merrick AE, Ilett EJ, Melcher AA (2009) JX-594, a targeted oncolytic poxvirus for the treatment of cancer. Curr Opin Investig Drugs 10: 1372–1382. 19943208

[7] Hwang TH, Moon A, Burke J, Ribas A, Stephenson J, et al (2011) A mechanistic proof-of-concept clinical trial with JX-594, a targeted multi-mechanistic oncolytic poxvirus, in patients with metastatic melanoma. Molecular Ther 19: 1913–1922. 10.1038/mt.2011.132 PMC3188739 21772252

[8] Breitbach CJ, Burke J, Jonker D, Stephenson J, Haas AR, et al (2011) Intravenous delivery of a multi-mechanistic cancer-targeted oncolytic poxvirus in humans. Nature 477: 99–102. 21886163 10.1038/nature10358

[9] Liu BL, Robinson M, Han ZQ, Branston RH, English C, et al (2003) ICP34.5 deleted herpes simplex virus with enhanced oncolytic, immune stimulating, and anti-tumour properties. Gene Ther 10: 292–303. 12595888 10.1038/sj.gt.3301885

[10] Kaufman HL, Kim DW, DeRaffele G, Mitcham J, Coffin RS, et al (2010) Local and distant immunity induced by intralesional vaccination with an oncolytic herpes virus encoding GM-CSF in patients with stage IIIc and IV melanoma. Ann Surg Oncol 17: 718–730. 19915919 10.1245/s10434-009-0809-6

[11] Kaufman HL, Bines SD (2010) OPTIM trial: a Phase III trial of an oncolytic herpes virus encoding GM-CSF for unresectable stage III or IV melanoma. Future Oncol 6: 941–949. 20528232 10.2217/fon.10.66

[12] Andtbacka RHI, Collichio FA, Amatruda T, Senzer NN, Chesney J, et al (2013) OPTiM: A randomized phase III trial of talimogene laherparepvec (T-VEC) versus subcutaneous (SC) granulocyte-macrophage colony-stimulating factor (GM-CSF) for the treatment (tx) of unresected stage IIIB/C and IV melanoma. J Clin Oncol 31: suppl abstr LBA9008.

[13] Campadelli-Fiume G, De Giovanni C, Gatta V, Nanni P, Lollini PL, et al (2011) Rethinking herpes simplex virus: the way to oncolytic agents. Rev Med Virol 21: 213–226. 21626603 10.1002/rmv.691

[14] Nishiyama Y, Kimura H, Daikoku T (1991) Complementary lethal invasion of the central nervous system by nonneuroinvasive herpes simplex virus types 1 and 2. J Virol 65: 4520–4524. 1649347 10.1128/jvi.65.8.4520-4524.1991 PMC248897

[15] Mori I, Liu B, Goshima F, Ito H, Koide N, et al (2005) HF10, an attenuated herpes simplex virus (HSV) type 1 clone, lacks neuroinvasiveness and protects mice against lethal challenge with HSV types 1 and 2. Microbes Infect 7: 1492–1500. 16054416 10.1016/j.micinf.2005.05.007

[16] Nakao A, Kimata H, Imai T, Kikumori T, Teshigahara O, et al (2004) Intratumoral injection of herpes simplex virus HF10 in recurrent breast cancer. Ann Oncol 15: 988–989. 15151960 10.1093/annonc/mdh225

[17] Fujimoto Y, Mizuno T, Sugiura S, Goshima F, Kohno S, et al (2006) Intratumoral injection of herpes simplex virus HF10 in recurrent head and neck squamous cell carcinoma. Acta Otolaryngol 126: 1115–1117. 16923721 10.1080/00016480600702100

[18] Kimata H, Imai T, Kikumori T, Teshigahara O, Nagasaka T, et al (2006) Pilot study of oncolytic viral therapy using mutant herpes simplex virus (HF10) against recurrent metastatic breast cancer. Ann Surgical Oncol 13: 1078–1084. 10.1245/ASO.2006.08.035 16865590

[19] Nakao A, Kasuya H, Sahin TT, Nomura N, Kanzaki A, et al (2011) A phase I dose-escalation clinical trial of intraoperative direct intratumoral injection of HF10 oncolytic virus in non-resectable patients with advanced pancreatic cancer. Cancer Gene Ther 18: 167–175. 21102422 10.1038/cgt.2010.65

[20] Kohno SI, Luo C, Nawa A, Fujimoto Y, Watanabe D, et al (2007) Oncolytic virotherapy with an HSV amplicon vector expressing granulocyte-macrophage colony-stimulating factor using the replication-competent HSV type 1 mutant HF10 as a helper virus. Cancer Gene Ther 14: 918–926. 17693992 10.1038/sj.cgt.7701070

[21] Goshima F, Esaki S, Luo C, Kamakura M, Kimura H, et al (2014) Oncolytic viral therapy with a combination of HF10, a herpes simplex virus type 1 variant and granulocyte-macrophage colony-stimulating factor for murine ovarian cancer. Int J Cancer 134: 2865–2877. 24265099 10.1002/ijc.28631

[22] Nocentini G, Giunchi L, Ronchetti S, Krausz LT, Bartoli A, et al (1997) A new member of the tumor necrosis factor/nerve growth factor receptor family inhibits T cell receptor-induced apoptosis. Proc Natl Acad Sci USA 94: 6216–6221. 9177197 10.1073/pnas.94.12.6216 PMC21029

[23] Pardoll DM (2012) The blockade of immune checkpoints in cancer immunotherapy. Nat Rev Cancer 12: 252–264. 22437870 10.1038/nrc3239 PMC4856023

[24] McHugh RS, Whitters MJ, Piccirillo CA, Young DA, Shevach EM, et al (2002) CD4(+)CD25(+) immunoregulatory T cells: gene expression analysis reveals a functional role for the glucocorticoid-induced TNF receptor. Immunity 16: 311–323. 11869690 10.1016/s1074-7613(02)00280-7

[25] Shimizu J, Yamazaki S, Takahashi T, Ishida Y, Sakaguchi S (2002) Stimulation of CD25(+)CD4(+) regulatory T cells through GITR breaks immunological self-tolerance. Nat Immunol 3: 135–142. 11812990 10.1038/ni759

[26] Cote AL, Zhang P, O'Sullivan JA, Jacobs VL, Clemis CR, et al (2011) Stimulation of the glucocorticoid-induced TNF receptor family-related receptor on CD8 T cells induces protective and high-avidity T cell responses to tumor-specific antigens. J Immunol 186: 275–283. 21106849 10.4049/jimmunol.1001308 PMC3050990

[27] Nishikawa H, Kato T, Hirayama M, Orito Y, Sato E, et al (2008) Regulatory T cell-resistant CD8+ T cells induced by glucocorticoid-induced tumor necrosis factor receptor signaling. Cancer Res 68: 5948–5954. 18632650 10.1158/0008-5472.CAN-07-5839

[28] Rosenzweig M, Ponte J, Apostolou I, Doty D, Guild J, et al (2010) Development of TRX518, an aglycosyl humanized monoclonal antibody (Mab) agonist of huGITR. J Clin Oncol 28: suppl abstr e13028.

[29] Takai T, Li M, Sylvestre D, Clynes R, Ravetch JV (1994) FcR gamma chain deletion results in pleiotrophic effector cell defects. Cell 76: 519–529. 8313472 10.1016/0092-8674(94)90115-5

[30] Lerner WA, Pearlstein E, Ambrogio C, Karpatkin S (1983) A new mechanism for tumor induced platelet aggregation. Comparison with mechanisms shared by other tumor with possible pharmacologic strategy toward prevention of metastases. Int J Cancer 31: 463–469. 6299977 10.1002/ijc.2910310411

[31] Muraoka D, Kato T, Wang L, Maeda Y, Noguchi T, et al (2010) Peptide vaccine induces enhanced tumor growth associated with apoptosis induction in CD8+ T cells. J Immunol 185: 3768–3776. 20733202 10.4049/jimmunol.0903649

[32] Ikeda H, Ohta N, Furukawa K, Miyazaki H, Wang L, et al (1997) Mutated mitogen-activated protein kinase: a tumor rejection antigen of mouse sarcoma. Proc Natl Acad Sci USA 94: 6375–6379. 9177225 10.1073/pnas.94.12.6375 PMC21057

[33] Huang AY, Gulden PH, Woods AS, Thomas MC, Tong CD, et al (1996) The immunodominant major histocompatibility complex class I-restricted antigen of a murine colon tumor derives from an endogenous retroviral gene product. Proc Natl Acad Sci USA 93: 9730–9735. 8790399 10.1073/pnas.93.18.9730 PMC38497

[34] Mitsui J, Nishikawa H, Muraoka D, Wang L, Noguchi T, et al (2010) Two distinct mechanisms of augmented antitumor activity by modulation of immunostimulatory/inhibitory signals. Clin Cancer Res 16: 2781–2791. 20460483 10.1158/1078-0432.CCR-09-3243

[35] Hamid O, Robert C, Daud A, Hodi FS, Hwu WJ, et al (2013) Safety and tumor responses with lambrolizumab (anti-PD-1) in melanoma. N Engl J Med 369: 134–144. 23724846 10.1056/NEJMoa1305133 PMC4126516

[36] Postow MA, Luke JJ, Bluth MJ, Ramaiya N, Panageas KS, et al (2013) Ipilimumab for patients with advanced mucosal melanoma. Oncologist 18: 726–732. 23716015 10.1634/theoncologist.2012-0464 PMC4063400

[37] Ribas A, Kefford R, Marshall MA, Punt CJ, Haanen JB, et al (2013) Phase III randomized clinical trial comparing tremelimumab with standard-of-care chemotherapy in patients with advanced melanoma. J Clin Oncol 31: 616–622. 23295794 10.1200/JCO.2012.44.6112 PMC4878048

[38] Weber JS, Dummer R, de Pril V, Lebbe C, Hodi FS (2013) Patterns of onset and resolution of immune-related adverse events of special interest with ipilimumab: detailed safety analysis from a phase 3 trial in patients with advanced melanoma. Cancer 119: 1675–1682. 23400564 10.1002/cncr.27969

[39] Ko K, Yamazaki S, Nakamura K, Nishioka T, Hirota K, et al (2005) Treatment of advanced tumors with agonistic anti-GITR mAb and its effects on tumor-infiltrating Foxp3+CD25+CD4+ regulatory T cells. J Exp Med 202: 885–891. 16186187 10.1084/jem.20050940 PMC2213162

[40] Robert C, Thomas L, Bondarenko I, O'Day S, Weber J, et al (2011) Ipilimumab plus dacarbazine for previously untreated metastatic melanoma. N Engl J Med 364: 2517–2526. 21639810 10.1056/NEJMoa1104621

[41] Brahmer JR, Tykodi SS, Chow LQ, Hwu WJ, Topalian SL, et al (2012) Safety and activity of anti-PD-L1 antibody in patients with advanced cancer. N Engl J Med 366: 2455–2465. 22658128 10.1056/NEJMoa1200694 PMC3563263

[42] Topalian SL, Hodi FS, Brahmer JR, Gettinger SN, Smith DC, et al (2012) Safety, activity, and immune correlates of anti-PD-1 antibody in cancer. N Engl J Med 366: 2443–2454. 22658127 10.1056/NEJMoa1200690 PMC3544539

[43] Curiel TJ, Coukos G, Zou L, Alvarez X, Cheng P, et al (2004) Specific recruitment of regulatory T cells in ovarian carcinoma fosters immune privilege and predicts reduced survival. Nat Med 10: 942–949. 15322536 10.1038/nm1093

[44] Wolf D, Wolf AM, Rumpold H, Fiegl H, Zeimet AG, et al (2005) The expression of the regulatory T cell-specific forkhead box transcription factor FoxP3 is associated with poor prognosis in ovarian cancer. Clin Cancer Res 11: 8326–8331. 16322292 10.1158/1078-0432.CCR-05-1244

[45] Wang W, Hodkinson P, McLaren F, MacKinnon A, Wallace W, et al (2012) Small cell lung cancer tumour cells induce regulatory T lymphocytes, and patient survival correlates negatively with FOXP3+ cells in tumour infiltrate. Int J Cancer 131: E928–937. 22532287 10.1002/ijc.27613

[46] Francisco LM, Salinas VH, Brown KE, Vanguri VK, Freeman GJ, et al (2009) PD-L1 regulates the development, maintenance, and function of induced regulatory T cells. J Exp Med 206: 3015–3029. 20008522 10.1084/jem.20090847 PMC2806460

[47] Simpson TR, Li F, Montalvo-Ortiz W, Sepulveda MA, Bergerhoff K, et al (2013) Fc-dependent depletion of tumor-infiltrating regulatory T cells co-defines the efficacy of anti-CTLA-4 therapy against melanoma. J Exp Med 210: 1695–1710. 23897981 10.1084/jem.20130579 PMC3754863

[48] Cohen AD, Diab A, Perales MA, Wolchok JD, Rizzuto G, et al (2006) Agonist anti-GITR antibody enhances vaccine-induced CD8(+) T-cell responses and tumor immunity. Cancer Res 66: 4904–4912. 16651447 10.1158/0008-5472.CAN-05-2813 PMC2242844

[49] Cohen AD, Schaer DA, Liu C, Li Y, Hirschhorn-Cymmerman D, et al (2010) Agonist anti-GITR monoclonal antibody induces melanoma tumor immunity in mice by altering regulatory T cell stability and intra-tumor accumulation. PLoS One 5: e10436. 20454651 10.1371/journal.pone.0010436 PMC2862699

[50] Li YQ, Liu FF, Zhang XM, Guo XJ, Ren MJ, et al (2013) Tumor secretion of CCL22 activates intratumoral Treg infiltration and is independent prognostic predictor of breast cancer. PLoS One 8: e76379. 24124553 10.1371/journal.pone.0076379 PMC3790712

[51] Imai N, Ikeda H, Tawara I, Wang L, Wang L, et al (2009) Glucocorticoid-induced tumor necrosis factor receptor stimulation enhances the multifunctionality of adoptively transferred tumor antigen-specific CD8+ T cells with tumor regression. Cancer Sci 100: 1317–1325. 19432889 10.1111/j.1349-7006.2009.01179.x PMC11158648

[52] La S, Kim E, Kwon B (2005) In vivo ligation of glucocorticoid-induced TNF receptor enhances the T-cell immunity to herpes simplex virus type 1. Exp Mol Med 37: 193–198. 16000873 10.1038/emm.2005.26

