# Supplementary figures and images for: Systemic CD8+ T Cell-Mediated Tumoricidal Effects by Intratumoral Treatment of Oncolytic Herpes Simplex Virus with the Agonistic Monoclonal Antibody for Murine Glucocorticoid-Induced Tumor Necrosis Factor Receptor
Source: PLoS One. 2014 Aug 8;9(8):e104669. doi: 10.1371/journal.pone.0104669 (PMC4126744; doi:10.1371/journal.pone.0104669)

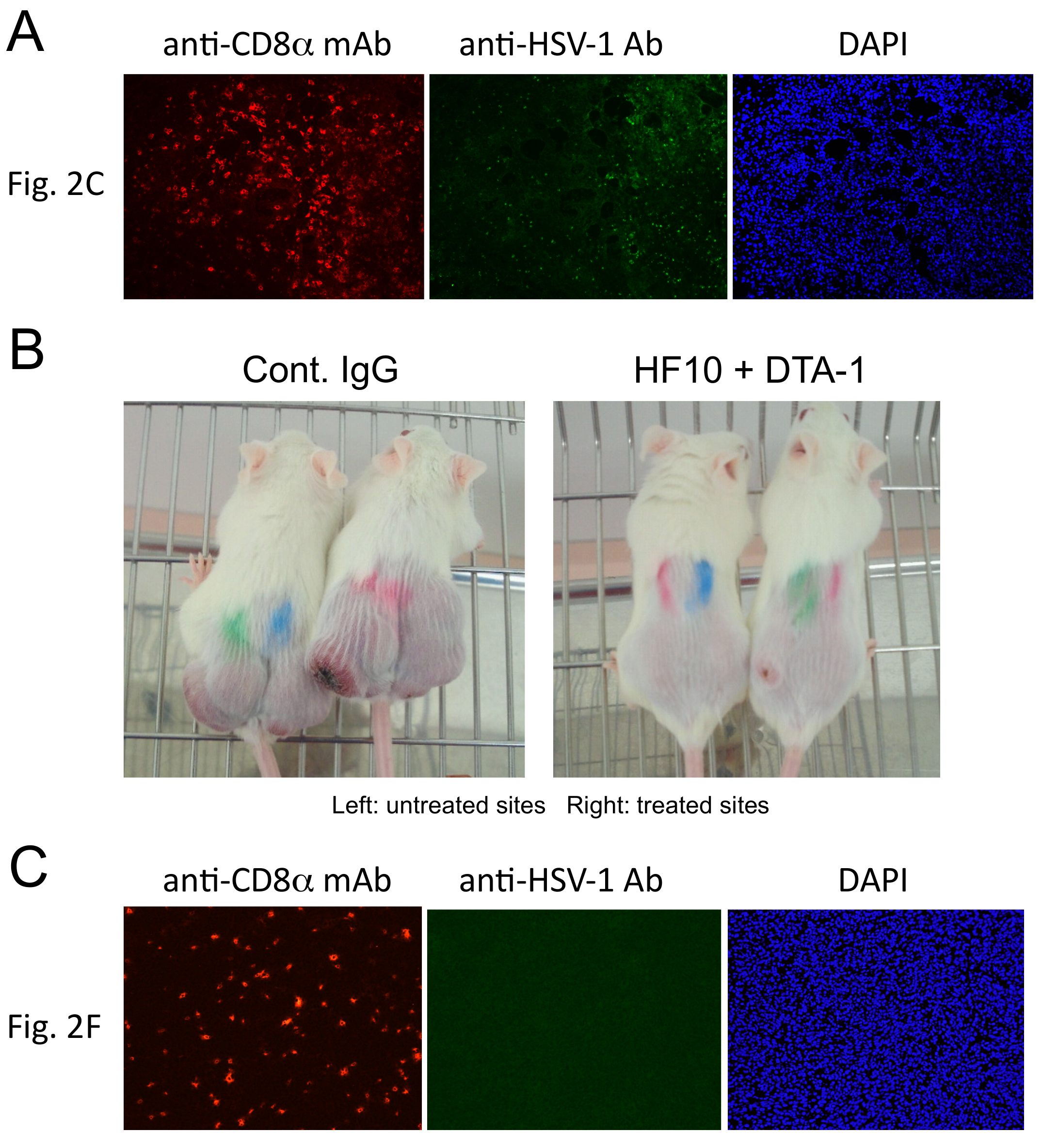

Supplement: Figure S1 — Systemic surveillance of tumoricidal CTLs after HF10 combination therapy with DTA-1 at local tumor sites. (A) The images of red (PE), green (FITC), and blue (DAPI) fluorescence that were merged to produce Fig. 2C. (B) Bilateral CT26/NY-ESO-1-bearing mice were treated i.t. with a combination of HF10 and DTA-1 in tumors on the right flanks of mice. Tumor growth in the treated right and contralateral left sites was measured. Photos show representative mice at 25 days after CT26/NY-ESO-1 inoculation from the control and dual HF10- and DTA-1-treated groups. (C) The three images of red (PE), green (FITC), and blue (DAPI) fluorescence that were merged to produce Fig. 2F. (TIF) [file pone.0104669.s001.tif]

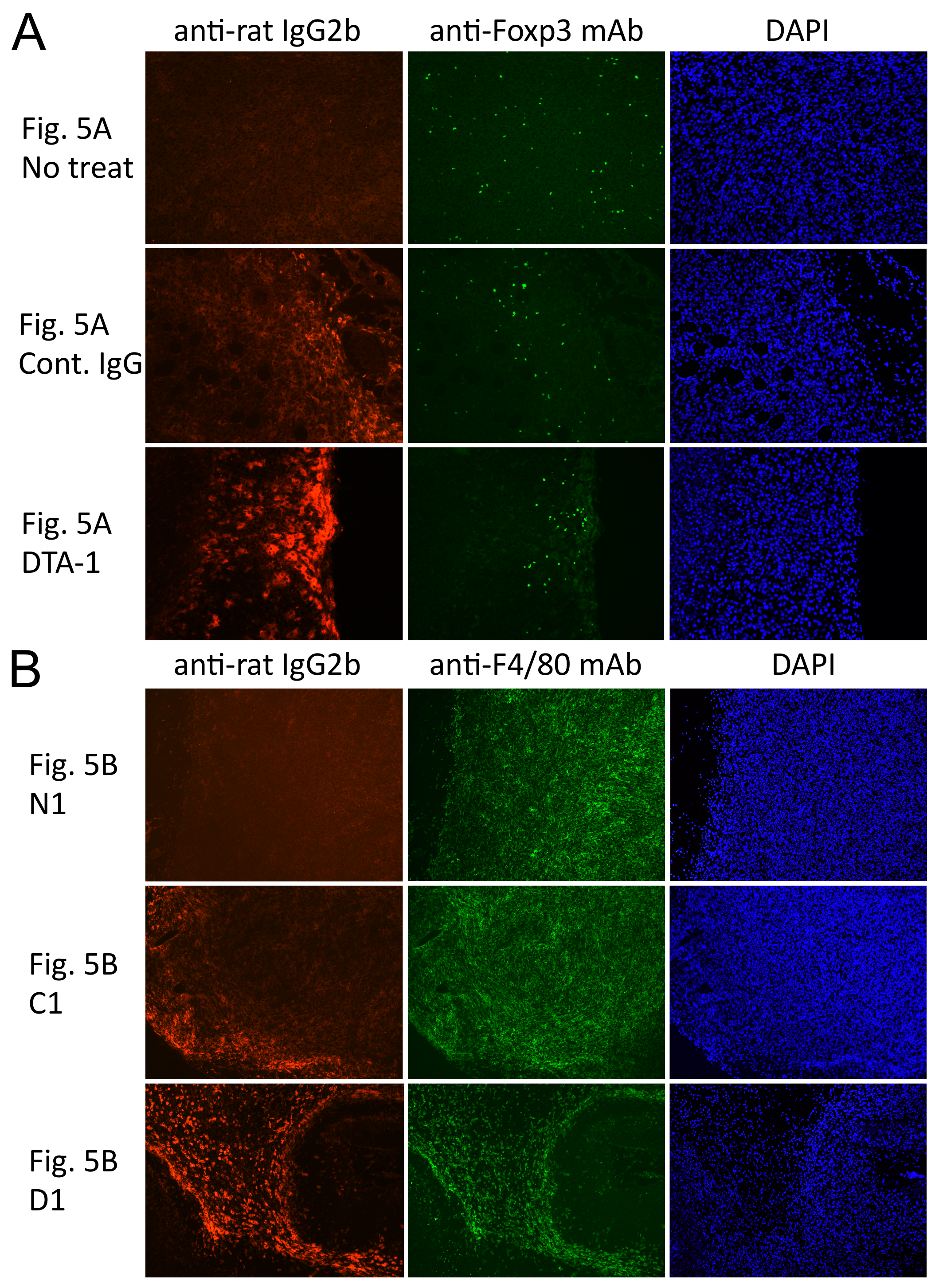

Supplement: Figure S2 — The three fluorescence components of the merged images of Fig. 5A and Fig. 5B (N1, C1, and D1). Three separate images of red (PE), green (FITC), and blue (DAPI) fluorescence that were merged to produce Fig. 5A (A) and N1, C1, and D1 of Fig. 5B (B). (TIF) [file pone.0104669.s002.tif]

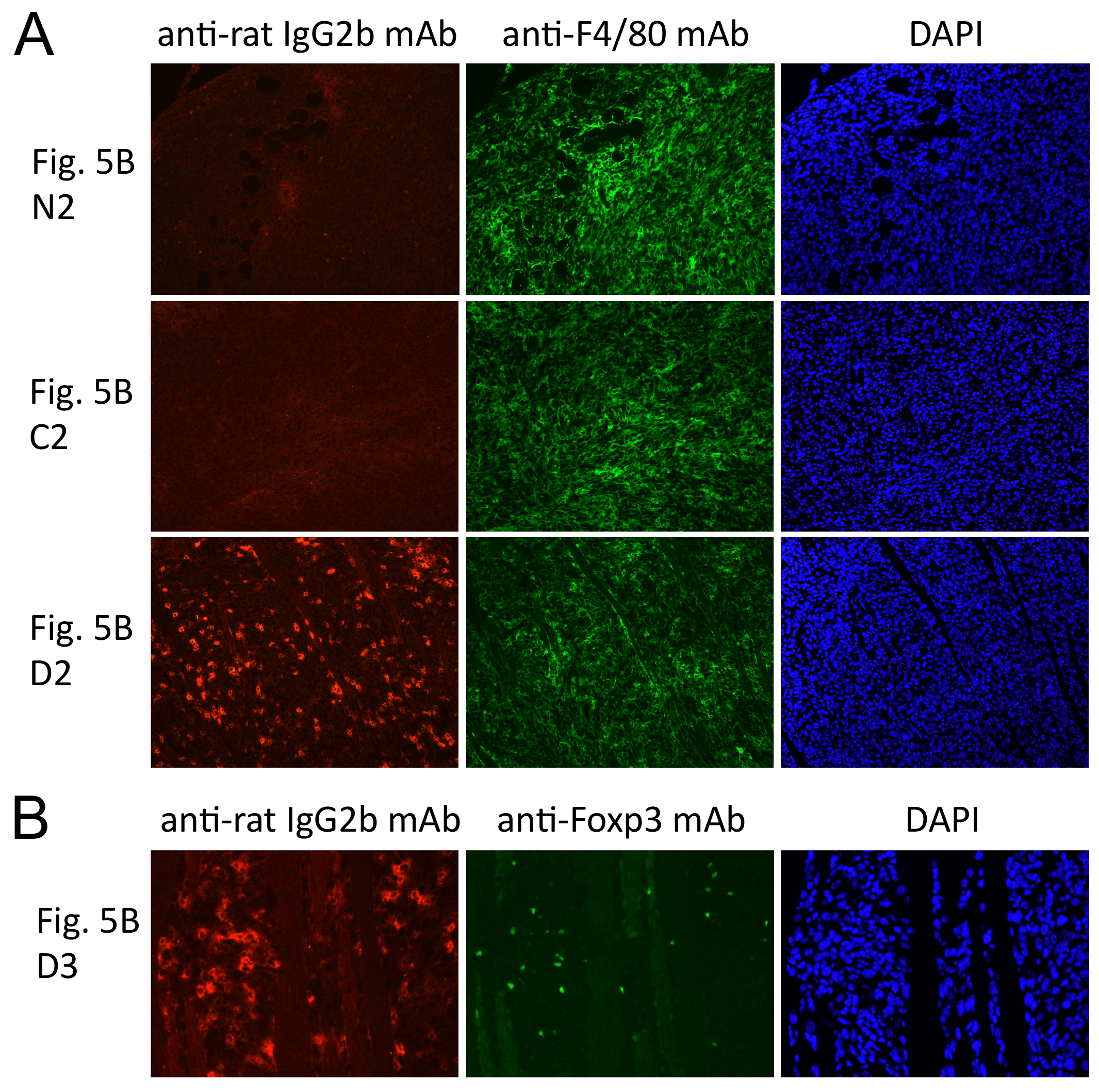

Supplement: Figure S3 — The three fluorescence components of the merged images of N2, C2, D2, and D3 in Fig. 5B . The three separate images of red (PE), green (FITC), and blue (DAPI) fluorescence that were merged to produce N2, C2, D2, and D3 images in Fig. 5B. (TIF) [file pone.0104669.s003.tif]

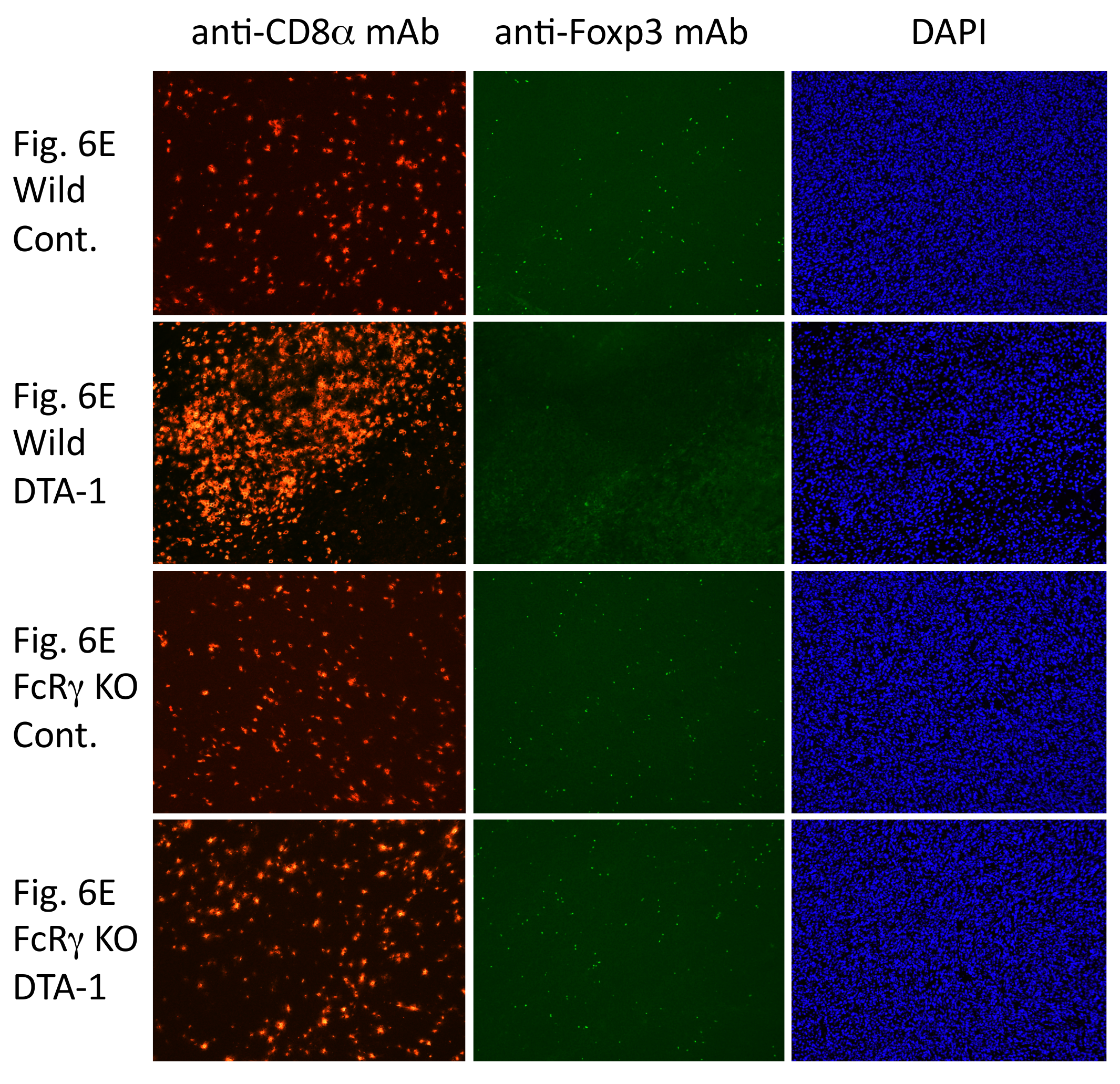

Supplement: Figure S4 — The three fluorescence components of the merged images in Fig. 6E . The three separate images of red (PE), green (FITC), and blue (DAPI) fluorescence that were merged to produce Fig. 6E. (TIF) [file pone.0104669.s004.tif]

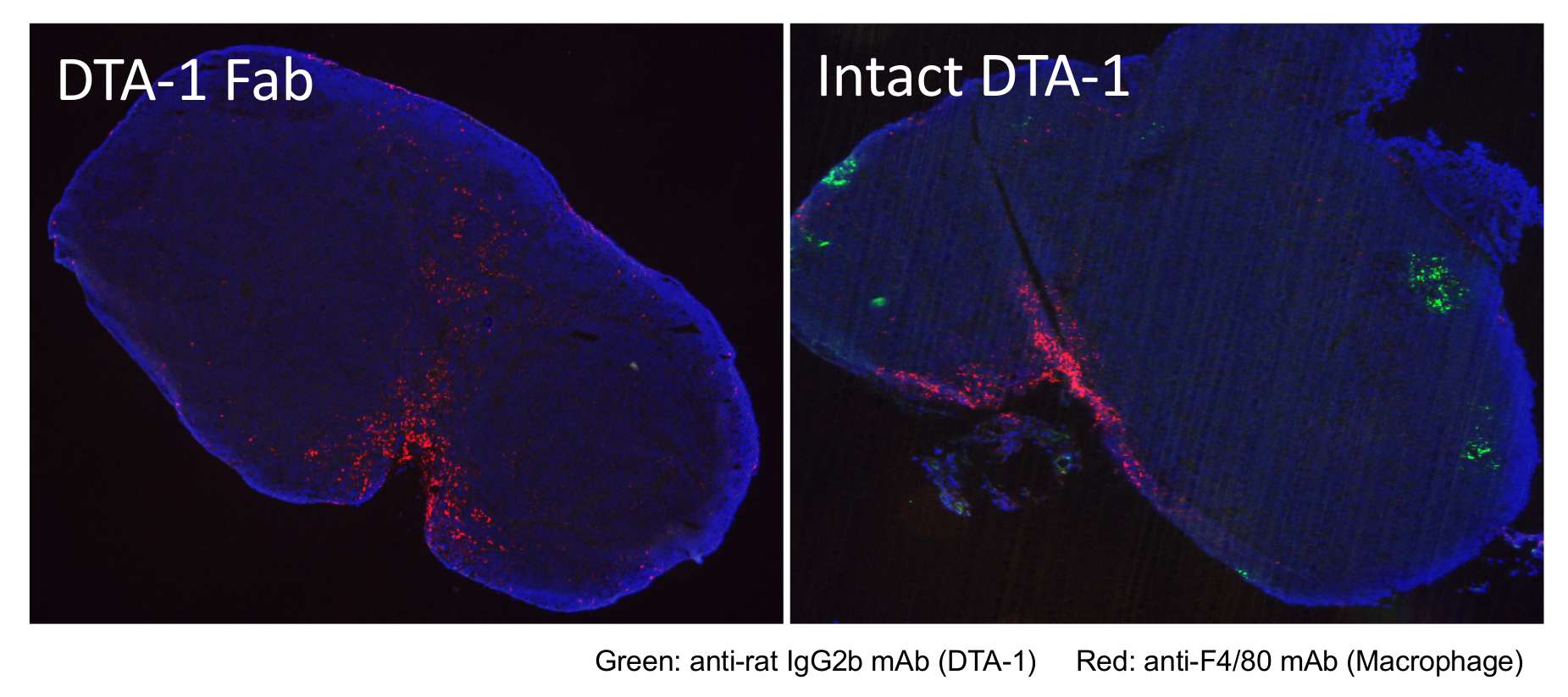

Supplement: Figure S5 — Drafting of i.t. treated DTA-1 into tumor-draining lymph nodes. Frozen sections of tumor-draining lymph nodes obtained at 6 hrs after intratumoral DTA-1 or DTA-1 Fab treatment were stained with a FITC-conjugated anti-rat IgG2b antibody, a phycoerythrin (PE)-conjugated anti-F4/80 antibody, and DAPI. (TIF) [file pone.0104669.s005.tif]
